# Splenic Surprise: Investigating a Case of Splenic Infarct as an Isolated COVID-19 Manifestation

**DOI:** 10.7759/cureus.53438

**Published:** 2024-02-02

**Authors:** Malina Mohtadi, Sacide S Ozgur, Joseph Russo, Nida Ansari, Patrick Michael

**Affiliations:** 1 Internal Medicine, St. Joseph's University Medical Center, Paterson, USA

**Keywords:** thrombosis, hypercoagulability, splenic infarct, sars-cov-2, covid-19

## Abstract

Coronavirus disease 2019 (COVID-19) infection has been associated with a multitude of complications, one established complication being thromboembolism, a result of the proinflammatory state induced by severe acute respiratory syndrome coronavirus 2 (SARS-CoV-2). This prothrombotic state is a cumulation of many inflammatory pathways at work. Here, we present an interesting case of a 43-year-old female who did not present with the typical COVID-19 clinical picture. Instead, she presented with periumbilical pain, nausea, and vomiting. Upon further investigation, she was found to have a splenic infarct on a computed tomography (CT) scan. An extensive workup was performed to explore possible etiologies; however, it was concluded that her splenic infarct was secondary to her COVID-19 infection. With this case, we aim to add to the literature regarding the manifestations of the prothrombotic state of SARS-CoV-2.

## Introduction

Coagulopathy is a well-known consequence of coronavirus disease 2019 (COVID-19) infection, manifesting as both venous and arterial thromboembolism [1]. When considering possible causes of splenic infarction, malignancy, cardiac origin, hematological disease, and infectious causes must also be assessed [2]. The prothrombotic state in the context of a COVID-19 infection is largely attributed to the widespread inflammation caused by severe acute respiratory syndrome coronavirus 2 (SARS-CoV-2) and is evident in markers such as lactate dehydrogenase and D-dimer [1]. Interestingly, a study in the Netherlands indicated that out of 184 ICU patients studied, only 3.7% of those with COVID-19 had arterial thrombosis [3]. However, upon literature review, it appears that splenic infarction due to COVID-19 is rarely reported, with no clear incidence documented. This case highlights not only rare sequelae of COVID-19 but also an abnormal presentation of splenic infarction.

## Case presentation

A 43-year-old female with a history of seasonal allergies presented to the emergency department (ED) with acute, crampy periumbilical pain, rated 9/10 in intensity, accompanied by nausea and vomiting. She reported that her daughter and mother had recently tested positive for COVID-19 but denied any personal history of abdominal trauma or recent travel.

Upon examination, her oxygen saturation was 97% on room air, blood pressure was 146/84 mmHg, heart rate was 82 bpm, and she was afebrile. An electrocardiogram (EKG) showed normal sinus rhythm with no ST or T wave changes. Laboratory investigations are detailed below in Table 1. A right upper quadrant ultrasound indicated a slightly fatty liver but was otherwise unremarkable. The patient tested positive for COVID-19, although she was asymptomatic for respiratory symptoms.

**Table 1:** Significant laboratory values observed upon admission

Laboratory tests	Values	Reference range
Hemoglobin	8.7 g/dL	12.0-16.0 g/dL
Mean corpuscular volume	64.0 fL	80.0-100 fL
Red cell distribution width	21.3%	0.5-16.5%
Iron level	11 mcg/dL	50-212 mcg/dL
Ferritin	16.0 ng/mL	14-233 ng/mL
Total iron binding capacity	414 mcg/dL	250-400 mcg/dL
Aspartate aminotransferase	16 U/L	13-39 U/L
Alanine aminotransferase	14 U/L	7-52 U/L
Lipase	35 U/L	11-82 U/L

**Figure 1:**
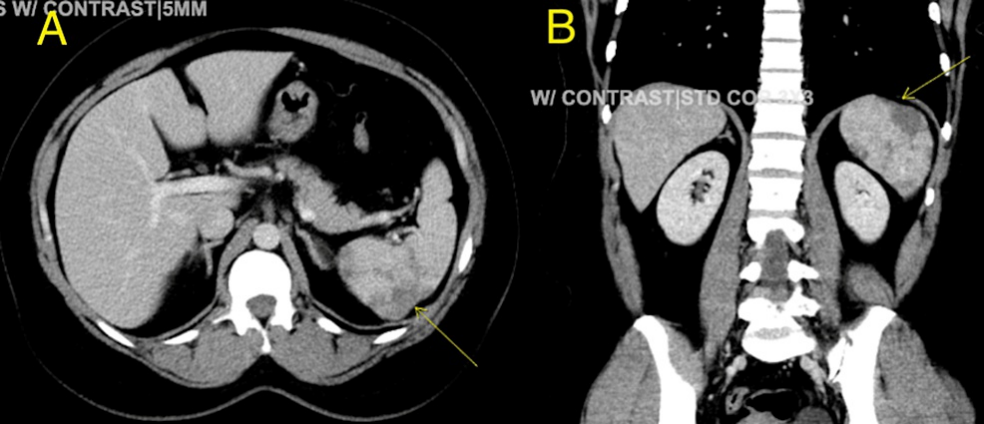
CT abdomen pelvis with contrast axial (A) and coronal (B) view showing splenic infarcts (yellow arrow)

A CT scan of the abdomen and pelvis with intravenous contrast revealed multiple areas of hypodensity in the spleen, consistent with splenic infarcts as seen in Figure 1A and B. The patient's medical history included a first-trimester spontaneous miscarriage and a family history of arterial thromboembolism (stroke and myocardial infarction in first-degree relatives in their 50s). Extensive testing, including Factor V Leiden, antiphospholipid antibody (Ab), lupus anticoagulant, anticardiolipin Ab, beta-2 glycoprotein Ab, Protein C and S, antithrombin III, paroxysmal nocturnal hemoglobinuria panel flow, haptoglobin, lactate dehydrogenase, hemoglobin electrophoresis, Coombs direct and indirect tests, and an autoimmune panel including ANA and Anti SSA/SSB Ab, were all within normal limits. JAK-2 mutation testing was later sent outpatient; however, results have not yet been obtained. A peripheral smear, or PCR was not obtained to rule out babesiosis. Blood cultures were negative. However, elevated D-dimer levels of 1.75 mcg/mL (normal range < 0.5) and Factor VIII levels of 411 (normal range 49-126) were noted. The patient was started on therapeutic enoxaparin 80 mg every 12 hours. An echocardiogram was performed to rule out a cardiac origin of arterial embolization, revealing a regular study with an ejection fraction of 60-65%, with no bubble study performed or further evaluation with a transesophageal echocardiogram. Upon discharge, she was prescribed apixaban 10 mg every 12 hours for one week, followed by 5 mg every 12 hours for six months, and ferrous sulfate for iron deficiency anemia. She followed up with her primary care doctor and remained asymptomatic.

## Discussion

At this time, the world is well aware of SARS-CoV-2 and the profound impact of the COVID-19 pandemic. However, understanding the pathophysiology of the coagulopathy it causes requires an understanding of its structure. Coronavirus is composed of four proteins; notably, the S spike allows for binding to the angiotensin-converting enzyme 2 (ACE2) receptor, predominantly found on cardiac myocytes, pulmonary type 2 pneumocytes, and vascular endothelial cells [4]. The binding of the S spike to the ACE2 receptor leads to a protease-mediated cleavage, enabling conformational changes of the S spike subunits for viral access and integration into host cells [5]. Upon entry and subsequent virus-induced apoptosis, involving the interaction of the E protein and a Bcl-2 protein, macrophages and dendritic cells phagocytose the cell and present its antigen [4,5]. This antigen presentation to the body’s T cells induces a widespread inflammatory response [4].

Inflammation and, in turn, coagulopathy in COVID-19 go hand in hand. Vascular endothelial damage from SARS-CoV-2 entry into endothelial cells via ACE2 receptors causes intracellular disruption and cellular swelling, leading to lymphocyte infiltration [4,6]. Macrophages and damaged endothelial cells expressing tissue factor (TF) and the involvement of interleukin-6 (IL-6) induce TF expression on infiltrating mononuclear cells [7]. TF induces a coagulopathic state by forming complexes with Factor VIIa, activating the extrinsic arm of the coagulation cascade [8]. Activated macrophages release pro-inflammatory cytokines like IL-6, IL-8, IL-10, and tumor necrosis factor α (TNF) [5], with IL-8 attracting neutrophils that enhance platelet clot formation [5]. This culmination of activity leads to a significantly prothrombotic state, resulting in thrombin and fibrin formation and clotting [4].

The presentation of this case is particularly interesting because splenic infarcts typically occur in patients with thromboembolic or hematological diseases [2]. Possible sources include infective endocarditis (IE), bacteremia without IE, malaria, babesia, or localized infections such as cellulitis [9]. A study reported that common symptoms in splenic infarction include left-sided abdominal pain (50%), nausea and/or vomiting (32%), fever above 38°C (36%), WBC count over 12,000 (56%), and elevated LDH (71%) [2]. Early-stage CT scans show a wedge-shaped infarct, while later stages require ultrasonography (US) to reveal hypoechoic abnormalities representing the infarct [2]. However, in this patient, the presentation included periumbilical pain rather than the typical left-sided abdominal pain, with no nausea, vomiting, palpation tenderness, or organomegaly. Her WBC count was 7,900, temperature was 36°C, and LDH was 239, all within normal limits. Fortunately, the CT scan of the abdomen revealed multiple hypodense areas of the spleen without focal fluid collection or adjacent induration, suggesting a splenic infarct, and the patent portal vein, superior mesenteric head, and splenic vein. The patient was evaluated by the surgical team and deemed to require no surgical intervention, leading to her discharge with enoxaparin and appropriate outpatient follow-up appointments.

## Conclusions

There is significant importance in highlighting cases such as this to raise awareness of the atypical presentation of splenic infarction, as well as to consider the patient’s risk factors, as observed in this patient who had an asymptomatic COVID-19 infection.

## References

[1] Abou-Ismail MY, Diamond A, Kapoor S, Arafah Y, Nayak L (2020). The hypercoagulable state in COVID-19: incidence, pathophysiology, and management. Thromb Res.

[2] Chapman J, Helm TA, Kahwaji CI (2024). Splenic infarcts. StatPearls [Internet].

[3] Klok FA, Kruip MJ, van der Meer NJ (2020). Incidence of thrombotic complications in critically ill ICU patients with COVID-19. Thromb Res.

[4] Kichloo A, Dettloff K, Aljadah M (2020). COVID-19 and hypercoagulability: a review. Clin Appl Thromb Hemost.

[5] Yuki K, Fujiogi M, Koutsogiannaki S (2020). COVID-19 pathophysiology: A review. Clin Immunol.

[6] Ackermann M, Verleden SE, Kuehnel M (2020). Pulmonary vascular endothelialitis, thrombosis, and angiogenesis in COVID-19. N Engl J Med.

[7] Levi M, Thachil J, Iba T, Levy JH (2020). Coagulation abnormalities and thrombosis in patients with COVID-19. Lancet Haematol.

[8] Butenas S, Orfeo T, Mann KG (2009). Tissue factor in coagulation: which? Where? When?. Arterioscler Thromb Vasc Biol.

[9] Im JH, Chung MH, Lee HJ, Kwon HY, Baek JH, Jang JH, Lee JS (2020). Splenic infarction and infectious diseases in Korea. BMC Infect Dis.

